# Ingestion of Alcohol-Based Hand Sanitizer: A case report

**DOI:** 10.1192/j.eurpsy.2022.2177

**Published:** 2022-09-01

**Authors:** T. Gutierrez Higueras, F. Calera Cortés, B. Hernández Gajate, E.D. Servin López, S. Sainz De La Cuesta Alonso, S. Vicent Forés

**Affiliations:** 1 Hospital Reina Sofía Córdoba, Psychiatry, Cordoba, Spain; 2 Reina Sofia University Hospital, Psychiatry, Córdoba, Spain; 3 Hospital Reina Sofía Córdoba, Psychiatry, Córdoba, Spain

**Keywords:** hand sanitizer, ingestion, emergency, Suicide

## Abstract

**Introduction:**

Alcohol-based hand sanitizers containing ethanol or isopropanol are being used in order to prevent person-to-person transmission during the COVID-19. Early signs and symptoms of this ingestion include nausea, vomiting, headache, abdominal pain, blurred vision, loss of coordination, and decreased level of consciousness. After hand sanitizer ingestion we have to suspect about methanol poisoning, monitoring the start of anion-gap metabolic acidosis, seizures, and blindness is essential. Treatment includes supportive care, acidosis correction, and the administration of an alcohol dehydrogenase inhibitor. In servere cases hemodialysis may be required.

**Objectives:**

To present a case of an 29-year-old woman who was taken to the emergency department after voluntary ingestion of alcohol-based hand sanitizer in a suicide attempt. To describe the most common side effects of hand sanitizer ingestion and the literature review.

**Methods:**

Clinical case presentation and literature review of similar cases.

**Results:**

A 29-year-old woman, with diagnosis of borderline personality disorder and previous suicide attempts was taken to the emergency department after 3 hours of voluntary ingestion of an unknown quantity of alcohol-based hand sanitizer. Initial laboratory findings showed laboratory a blood methanol concentration of 66 mg/dL, with an anion gap of 30 mEq/L, arterial blood pH of 7.2, serum bicarbonate concentration of 12 mEq/L. Patient complained of abdominal pain and nervoussness.

**Conclusions:**

Most common signs and symptoms of alcohol-based hand sanitizer ingestion include nausea, vomiting, headache, abdominal pain, blurred vision, loss of coordination, and decreased level of consciousness. Treatment includes supportive care, acidosis correction, the administration of an alcohol dehydrogenase inhibitor and sometimes may be required.

**Disclosure:**

No significant relationships.

